# Prioritising neonatal medicines research: UK Medicines for Children Research Network scoping survey

**DOI:** 10.1186/1471-2431-9-50

**Published:** 2009-08-12

**Authors:** Mark A Turner, Sara Lewis, Daniel B Hawcutt, D Field

**Affiliations:** 1 Division of Perinatal and Reproductive Medicine, University of Liverpool, Liverpool, UK; 2 National Perinatal Epidemiology Unit, University of Oxford, Oxford, UK; 3 Department of Health Sciences, University of Leicester, Leicester, UK

## Abstract

**Background:**

The dosing regimen and indications for many medicines in current use in neonatology are not well defined. There is a need to prioritise research in this area, but currently there is little information about which drugs are used in UK neonatal units and the research needs in this area as perceived by UK neonatologists.

**Methods:**

The Neonatal Clinical Studies Group (CSG) of the Medicines for Children Research Network (MCRN) undertook a 2 week prospective scoping survey study to establish which medicines are used in UK neonatal units; how many babies are receiving them; and what clinicians (and other health professionals) believe are important issues for future research.

**Results:**

49 out of 116 units responded to at least one element of the survey (42%). 37 units reported the number of neonates who received medicines over a 2 week period. A total of 3924 medicine-patient pairs were reported with 119 different medicines. 70% of medicine-patient pairs involved medicines that were missing either a license or dose for either term or preterm neonates. 4.3% of medicine-patient pairs involved medicines that were missing both license and dose for any neonate. The most common therapeutic gap in need of additional research identified by UK neonatologists was chronic lung disease (21 responding units), followed by patent ductus arteriosus and vitamin supplements (11 responding units for both)

**Conclusion:**

The research agenda for neonatal medicines can be informed by knowledge of current medicine use and the collective views of the neonatal community.

## Background

At present there are several drivers for research about medicines for neonates. The dosing regimen and indications for many medications in current use are not well defined. Up to 90% of babies receiving medication on a neonatal unit receive unlicensed or off label drugs [1]. The recent European Union Regulation on Medicines for Paediatric Use requires studies in neonates to be included in licensing applications for medicines that could be used in the newborn. This will increase the number of trials in the newborn. There is a relatively small number of infants, particularly at gestational ages < 29 weeks. This leads to a need to prioritise research in this area, so that high quality research is carried out to answer important clinical questions from finite funding opportunities. The process of prioritisation involves an assessment of disease burden and available medicines, as exemplified by the work done by the European Medicines Agency (EMEA) for off-patent medicines. This should be coupled with assessments of the pattern of medicines use and the licensing status of medicines [2,3]. Surveys of medication use in neonatal medicine have been performed before but not recently in UK.

The Neonatal Clinical Studies Group (CSG) of the Medicines for Children Research Network (MCRN) has a remit to develop the UK portfolio of neonatal medicines research. Part of this portfolio will relate to existing medicines. The CSG decided to start its portfolio development work with an examination of medicines use in UK neonatal units. The MCRN set up an Extended Neonatal Network (ENN) to support the development and delivery of the portfolio. The aims of this 2 week prospective scoping survey study were to examine whether the Extended Neonatal Network of the MCRN could be used: i) to establish which medicines are used in UK neonatal units, and their relative frequency of use; ii) to determine what clinicians (and other health professionals) believe are important issues for future research.

## Methods

A scoping survey was devised by the authors (MT, SL, and DF) and revised by members of the Neonatal and Pharmacy CSGs of the MCRN. The survey was circulated by e-mail to 116 neonatal units that are members of the MCRN Extended Neonatal Network (ENN) and similar groups in the devolved nations. Two reminders were sent by e-mail. The two week data collection period occurred at the convenience of each Unit (December 2007 - April 2008). The units were characterised by the level of care that they provide and their location within the UK. Medicines were grouped by clinical indications. Each unit counted the number of neonates who received each medicine giving a number of medicine-patient pairs.

The nature of future research will depend on which information is currently not available to prescribers. In the UK the standard source of dosing information is the British National Formulary for Children (BNFC). This well-established formulary meets WHO standards for national formularies and is based on a broad range of expert opinion. The BNFC provides dosage information and indicates when information about medicine is not supported by a marketing authorisation, what the BNFC describes as "not licensed". BNFC 2007 was used to determine whether a medicine was "not licensed", and as a reference for doses, since it was the edition available to prescribers at the time of the survey. Medicine-patient pairs were classified according to the presence or absence of each of the following criteria: licensed in preterm neonates; licensed in term neonates; dose provided for preterm neonates; dose provided for term neonates. Blood products were excluded from the survey. Intravenous electrolyte supplements and parenteral nutrition were reported inconsistently and not included in the results.

Therapeutic gaps identified by respondents were classified by two investigators (MT, DH) and any differences resolved by discussion.

This survey was conducted as a service evaluation and so did not require consideration by a Research Ethics Committee: this decision was made by the authors and we did not seek the opinion of an Ethics Committee on this point.

## Results

### Drugs Prescribed on Neonatal Units

Of the 116 units contacted, 45 units indicated which medicines they prescribed over a 2 week period with 37 reporting how many babies received each medicine during a 2 week period. The level of care provided by each unit and its location within the UK are given in Table 1 for responders and non-responders. The characteristics did not differ between responders and non-responders (chi-squared test, not significant). In total 3924 medicine-patient pairs were reported by the units, using 119 different medicines. The drugs prescribed to the greatest number of neonates were gentamicin (n = 417), benzylpenicillin (n = 350), vitamin K (n = 332), caffeine (n = 249) and dalivit (n = 242). 28% of medicine-patient pairs over the 2 weeks survey period had complete information available, i.e. licenses for use in term and preterm neonates with doses for both groups (Table 2). On the other hand 4.3% of medicine-patient pairs lacked both licences and doses in both term and preterm neonates (Table 2). This latter group included medicines such as chlorhexidine (n = 95) and dexamethasone (n = 11). Excluding medicines whose license status was unclear (including heparin, hydrocortisone, phenytoin), a total of 70% of medicine-patient pairs had incomplete data for at least one criterion (Table 2).

**Table 1:** Characteristics of units that did and did not respond to the survey.

Responder status	Level of care provided by unit				Total
	1	2		3	
Non-Responders	13 (73)	21 (64)		45 (69)	79 (68)
Responders	5 (27)	12 (36)		20 (31)	37 (32)

Responder status	Nation within UK				Total
	England	Scotland	Wales	Northern Ireland	
Non-Responders	57 (67)	12 (80)	8 (73)	2 (40)	79 (68)
Responders	28 (33)	3 (20)	3 (27)	3 (60)	37 (32)

Each cell includes the number of units in that category and the percentage of responders and non-responders for that category. *Level 1 *Units provide Special Care but do not aim to provide any continuing High Dependency or Intensive Care. This term includes units with or without resident medical staff. *Level 2 *Units provide High Dependency Care and some short-term Intensive Care. *Level 3 *Units provide the whole range of medical neonatal care but not necessarily all specialist services such as neonatal surgery.

**Table 2:** Medicine-patient pairs classified according to information given in the BNFC 2007 about licensing status and dosage.

Licensing and dosage status according to BNFC 2007				Number of medication-	% of Total
Licensed in preterm	Licensed in term	Dose in preterm	Dose in term	patient pairs in each category	Medication-patient pairs
No	No	No	No	167	4.3
No	No	No	Yes	697	18
No	No	Yes	Yes	242	6.2
No	Yes	No	Yes	50	1.3
Yes	Yes	No	No	388	9.9
Yes	Yes	No	Yes	1174	30
Yes	Yes	Yes	Yes	1115	28
Unclear	Unclear	-	-	91	2.3
			Total	3924	100

### Therapeutic Gaps Identified

**Figure 1:**
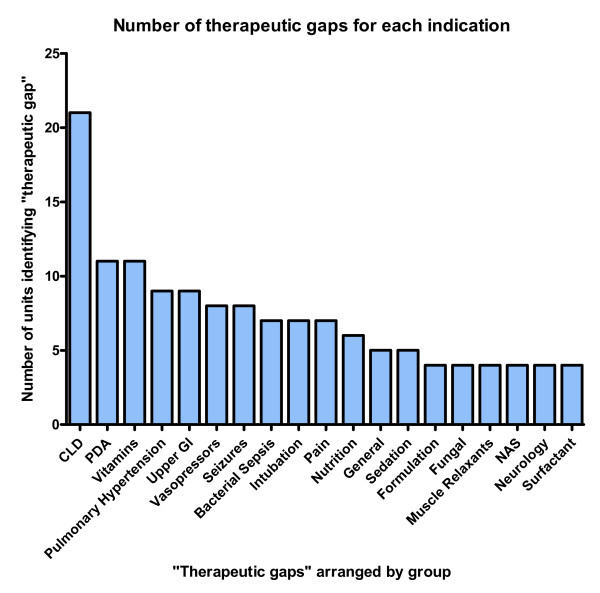
**Number of therapeutic gaps for each indication**. Graph of groups of identified therapeutic gaps against number of units who identified them. The abbreviations are: CLD, chronic lung disease; PDA, patent ductus arteriosus; GI, Gastrointestinal; NAS, neonatal abstinence syndrome.

37 units suggested one or more therapeutic gaps, providing 162 suggestions in total. Therapeutic gaps were grouped by indication and are shown in Figure 1. The most common therapeutic gap was chronic lung disease (raised by 21 responding units), followed by patent ductus arteriosus and vitamin supplements (n = 11 for both). Full results for all aspects of the survey are available from the Extended Neonatal Network on request sara.lewis@npeu.ox.ac.uk.

## Discussion

The results of this survey are comparable with recent results from the USA [4]. For example the most frequently prescribed medications on neonatal units in the USA were ampicillin and gentamicin [4]. 28% of the medicine-patient pairs involved medicines that were reported to have licenses and doses for both term and preterm neonates. This represents a high number of prescriptions for a relatively small number of medicines (mainly benzyl penicillin and gentamicin). The fact that 70% of medicine-patient pairs reported during the scoping exercise involved medicines that were lacking at least one piece of information suggests that there is a substantial research agenda in this area.

The methodology used here is simple, was acceptable to a range of neonatal units and provides a novel approach to estimating the impact of licensing status. The ENN provides a useful way to access UK neonatologists. One approach to assessing the impact of licensing status is to count the number of licences for neonatal medicines. For example among the new molecular entities licensed for use in a paediatric population in the US between 1998 and 2002 only 2% were licensed for use in the newborn period [5]. Our methodology extends this approach by estimating the extent of medicines use in neonates that falls under a marketing authorisation and the extent to which medicines use is not covered by a marketing authorisation. A repeat survey in several years time would indicate how changes in the regulatory framework, such as the EU regulation, have impacted on patients.

Clinicians are aware of gaps in the therapies they can offer. This will help inform the future direction of neonatal pharmacological research. One exemplar is the "The Newborn Drug Development Initiative", which was organised in the USA by the FDA and NIH Institute for Child Health and Human Development. This collaboration developed a process for deciding which drugs were most in need of study in this population (taking account of the nature of disease, outcome, drug characteristics, feasibility, methodology and ethics) [6]. Based on this work, criteria have been established for the investigation of a drug in the US neonatal population. The information we have gathered could be used in a similar dialogue between different stakeholders (e.g. parents, professionals, funders and regulators).

The limitations of this work include the incomplete response rate. This arose due to the lack of time among busy professionals to complete an unfunded survey. We acknowledge that the units who did respond may potentially represent a biased subset of units. However, we believe that the response rate was sufficient to yield a representative range of medicines used in this patient group. Our estimate of the extent of medicines use that lacks information relevant to prescribers is likely to reflect the true number of medicines requiring more information. We are also aware that the BNFC is not the only source of information about licensing status and dosing available. However, the study was designed and carried out through the UK's MCRN and the BNFC is well recognised, widely available, and is the national formulary of UK MCRN units. Comparisons with other sources would have detracted from our aim of contributing to UK prioritisation. The denominator in this study is the number of medicine-patient pairs on a neonatal unit over a 2 week period. The 2 week period varied between units. The number of units, an increased response rate due to the flexibility of the design and the random nature of admissions to neonatal units are likely to have outweighed the potential for bias introduced by the lack of a standardised time point for the start of data collection. Future work could include an estimate of the number of neonates. However, that would introduce the need to standardise for variation between units in admission criteria and for variation in the gestational age profile.

We have presented a summary of the data. The results also indicate which units use which medicines and the likely numbers of neonates in each unit who use each medicine. This information will facilitate research by allowing the rapid identification of units who might be interested in a particular research project. We anticipate that similar studies will be conducted by national bodies across Europe. These studies will facilitate international cooperation in areas of common need as well as national prioritisation.

## Conclusion

To date, the agenda about research into medicines for neonates has been informed by the interests of individual investigators and funded on the basis of the quality of individual trials. Our results raise the possibility that the research agenda can also be informed by the extent of medication use and the collective views of the neonatal community. We speculate that this will facilitate rational priority setting by directing the community towards neglected areas (such as Vitamin supplements) and be relevant to decisions about funding and collaboration at national and international levels.

## Competing interests

DF was Chair of the Neonatal CSG when the survey was done and MT is the Chair at the time of submission. SL is the co-ordinator of the ENN.

## Authors' contributions

MAT, SL and DF devised the scoping study and collated the results.

DH contributed to analysis and drafted and revised the manuscript in conjunction with MAT, SL and DF. All authors read and approved the final version of the manuscript.

## Pre-publication history

The pre-publication history for this paper can be accessed here:


